# Large drainage systems produced half of Mars’ ancient river sediment

**DOI:** 10.1073/pnas.2514527122

**Published:** 2025-11-24

**Authors:** Abdallah S. Zaki, Timothy A. Goudge, David Mohrig

**Affiliations:** ^a^Department of Earth and Planetary Sciences, Jackson School of Geosciences, The University of Texas at Austin, Austin, TX 78712; ^b^Center for Planetary Systems Habitability, The University of Texas at Austin, Austin, TX 78712

**Keywords:** Mars, drainage systems, sedimentary basins

## Abstract

Large, continental-scale drainage systems occupy nearly half of Earth’s land, shaping diverse ecosystems, with their activity primarily driven by climate and tectonism. Here, we investigate whether Mars, which lacks Earth’s tectonism, has similarly large drainage systems that could have impacted the extent of habitable environments, sediment transport, and the formation of large sedimentary basins. We reconstruct large drainage systems using globally mapped ancient martian water-formed topography—valley networks, outlet canyons, and fluvial depositional systems—revealing large drainage systems (>10^5^ km^2^) similar in scale to those on Earth. However, collectively these systems cover only ~5% of ancient martian terrain (>3.7 Ga), approximately nine times less than their counterparts on Earth. This estimate is conservative due to erosion, burial, and impact cratering, which have likely modified their preservation. Nevertheless, when quantifying the eroded sediment volumes from these large drainage systems, we show that they contributed nearly half (42%) of Mars’ ancient river-sediment budget. We further calculated the sediment eroded from large drainage systems surrounding the largest known fluvial depositional landforms on Mars and found that they potentially contributed ~21% of the planet’s river sediment budget, which we hypothesize facilitated the buildup of these sedimentary features. Our results indicate that although a substantial portion of Mars’ fluvial sediment was routed through large-scale drainage systems, sediment transport was predominantly accomplished within smaller-scale local basins. This drainage structure likely created a mosaic of habitable environments across the surface and facilitated significant sediment accumulation in a limited number of large sedimentary basins.

## Significance

Rivers on Earth are large and dynamic systems that diversify ecosystems through interactions between climate and tectonics. Here, we show that even in the absence of plate tectonics on Mars, large drainage systems existed; however, they are far less abundant on the landscape, occupying only ~5% of ancient martian terrain, suggesting Mars’ river history was predominantly influenced by smaller, local catchments. Yet, despite this small area coverage, large drainage systems account for nearly half of the planet’s total river sediment budget. The sediment from these drainage systems likely accumulated in extensive sedimentary basins. These findings have important implications for understanding habitable environments and the formation of large sedimentary basins on early Mars.

**Fig. 1.**
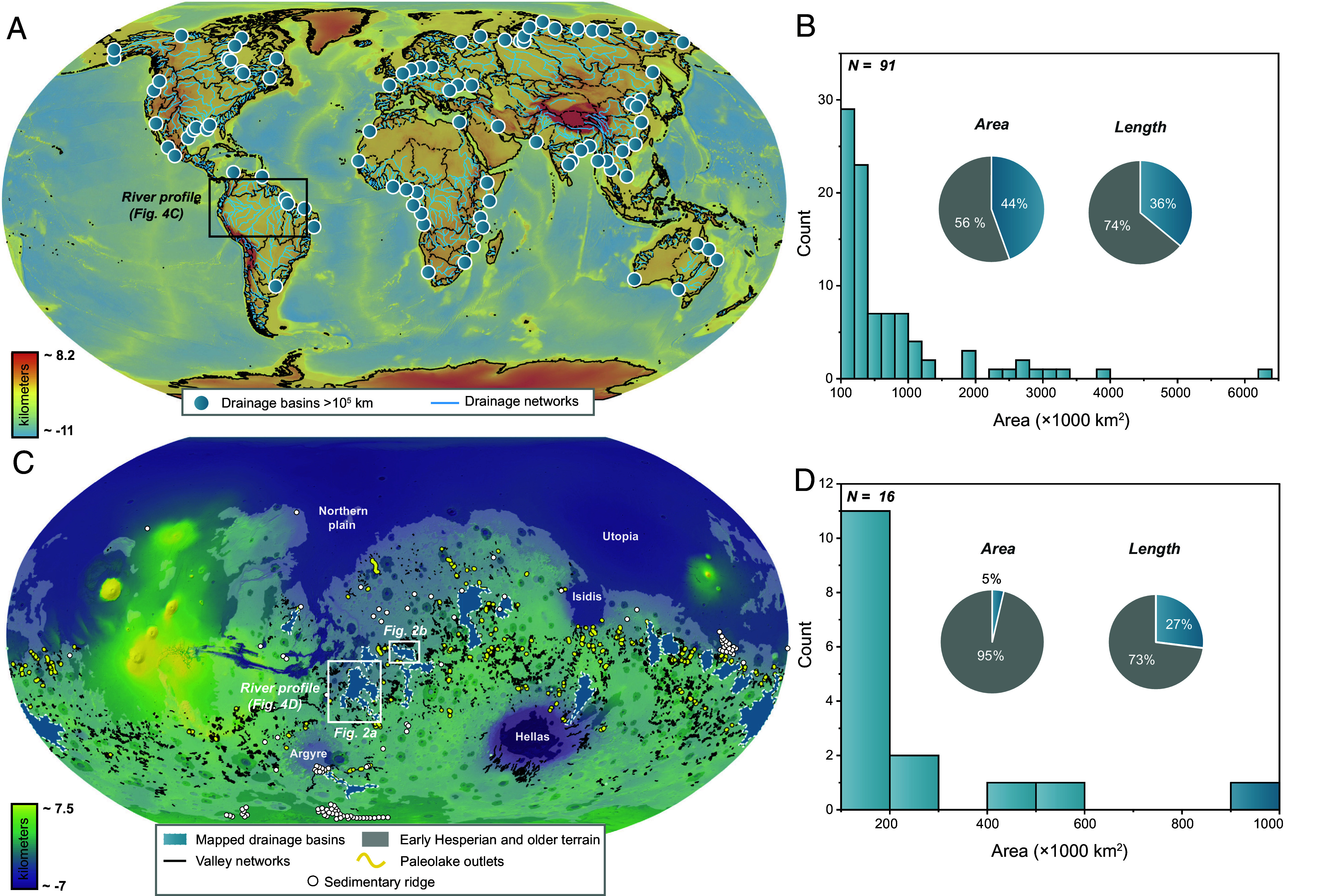
Global distribution of large drainage systems on Earth and Mars, and their characteristics. (*A*) Distribution of outlets for 91 drainage systems on Earth exceeding 10^5^ km^2^ (5). (*B*) Histogram of large drainage system areas on Earth, with inset pie charts showing proportions of total area and length contributed by large drainage systems (blue) compared to smaller systems (gray) (5). (*C*) Distribution of the 16 mapped large drainage systems on Mars. (*D*) Histogram of large drainage system areas on Mars, with inset pie charts illustrating the proportion of Early Hesperian and older terrains occupied by large drainage systems (blue) versus those not (gray), and the proportion of total valley network length represented by valleys within large drainage systems (blue) versus those outside these systems (gray). The background map of Earth is derived from the ETOPO Global Relief Model (7), while the background map of Earth is based on Mars Orbiter Laser Altimeter (MOLA) topography (8).

Large drainage systems on Earth are major components of continental landscapes, shaping some of the planet’s most diverse ecosystems and serving as the cradle of ancient and modern civilizations (1–3). These rivers have likely played a key role in regulating water–atmosphere interactions over geologic timescales by influencing biogeochemical processes (1, 4). Furthermore, modern large drainage systems transport global-scale sediment and solute fluxes (5). Global datasets of river networks and fluxes identify 91 “large” drainage systems on Earth, classified as those with drainage areas exceeding 10^5^ km^2^ (5). These drainage systems collectively occupy approximately 44% of Earth’s global land area and contribute over ~80% of total dissolved solids and ~70% of total suspended solids transported to the oceans (Fig. 1 *A* and *B*, 5). These estimates are consistent with recent findings indicating that Earth’s largest 25 rivers account for more than half of the total sediment flux transported to the oceans (6).

The origin, evolution, and physiography of these large drainage systems are primarily governed by plate tectonics (1, 9–11), the primary mechanism of generating relief on Earth. The tectonics at plate boundaries uplift mountain ranges, forming river sources, while subsidence creates extensive lowland basins that accommodate downstream river systems. Under sufficiently wet climates, precipitation-driven erosion reshapes upstream landscapes and deposits sediment downstream (9–11). This unique topographic setting raises the question of whether a planet that once had sustained available surface water but did not have plate tectonics would still form large drainage systems? Addressing this fundamental question not only illuminates the role of tectonism in shaping large drainage systems but also highlights how hydroclimate might shape the landscapes of other rocky worlds.

Mars is a planet without plate tectonics that preserves evidence of diverse, ancient, water-formed landscapes, including valley networks (12–14), outlet canyons (15–17), depositional rivers (18–21), lakes (22–24), and fan-shaped landforms (25, 26). The valley networks dissected the southern highlands, often filling craters with water to the point of breaching-generated flooding that formed extensive outlet canyons spanning hundreds of kilometers (16, 17, 24). The concentration of these channelized forms is higher on Early Hesperian and older terrains (>3.7 Ga), highlighting the peak period of valley incision (13, 14, 21, 23, 27). This period of valley incision followed an earlier era of more widespread landscape degradation (13, 23, 28, 29). In addition to incised valleys near the northern lowlands, along the martian hemispheric dichotomy, two major sites—Aeolis Dorsa and Arabia Terra—preserve ridges interpreted as depositional rivers (20, 21). The presence of these fragmentary sedimentary archives, when pieced together, could be fundamental in determining whether Mars once hosted large drainage systems comparable to those on Earth. If such rivers existed, how much sediment did they contribute to Mars’ global sediment budget? What role did they play in building up large martian sedimentary basins?

Here, we address these questions to understand whether Mars, which lacks plate tectonics, developed large drainage systems. To accomplish this objective, we combine the global distribution of previously mapped valley networks (12–14), outlet canyons (15–17), lakes (22–24), and depositional rivers (17–20). We then manually map potential drainage basins encompassing these landforms using modern topography. However, we note that this likely represents a conservative estimate of drainage area, as present-day topography does not completely reflect paleotopography at the time of valley network incision. For this reason, automated watershed delineation was not used, as such methods could introduce significant misinterpretations. Next, we quantify the volumes of sediment eroded from these mapped basins using a previously compiled dataset of valley depth (17, 30), and compare them to the global sediment budget from all valley systems (*Materials and Methods*). Based on our analysis and interpretations, we infer the mechanisms that shaped the formation of large drainage systems on Mars. Our findings have implications for determining whether Mars’ fluvial history was dominated by regional or local drainage systems and for understanding the development of its large sedimentary basins and associated habitability.

## Results

### Global Mapping of Large Drainage Systems on Mars.

**Fig. 2.**
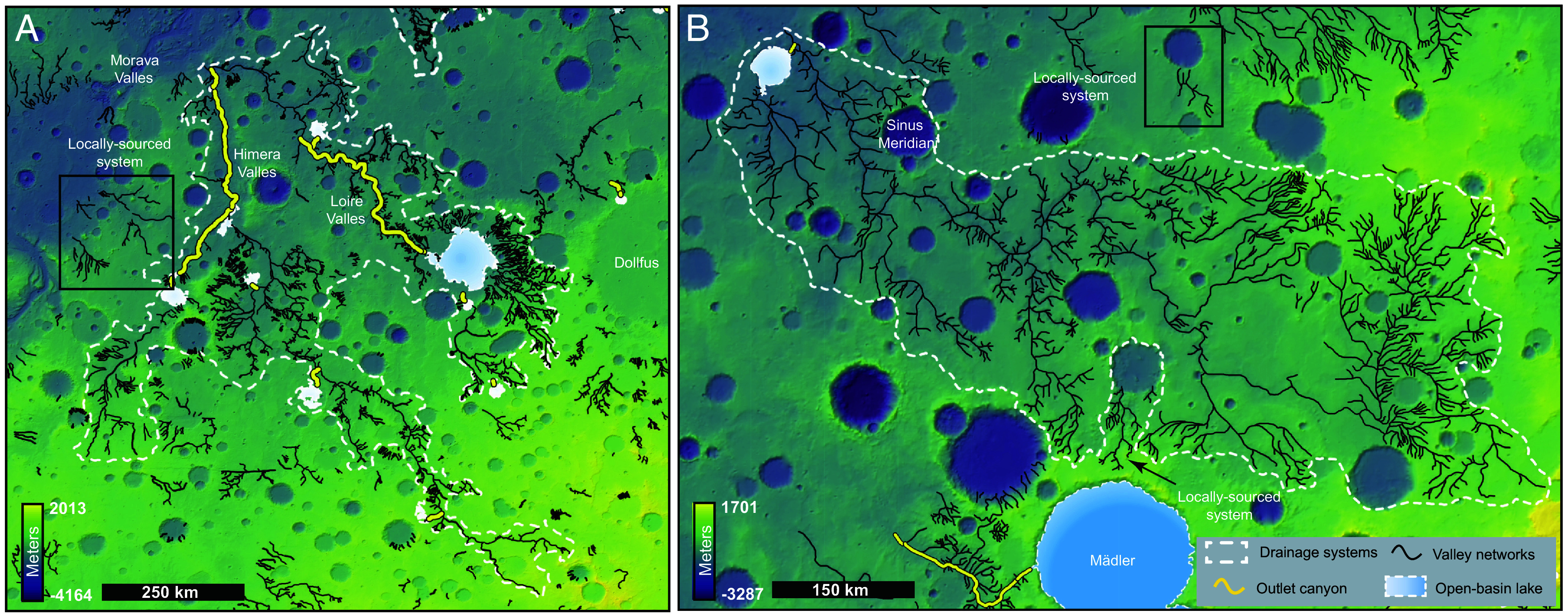
Examples of mapped large drainage systems on Mars. The dotted white outlines represent drainage boundaries encompassing valley networks (black), lakes (gradient blue), and outlet canyons (yellow) formed by lake breaches. The background map is derived from MOLA topography. (*A*) Large drainage System 1 centered at –22.4°N, –17.7°E, with substantial contributions from outlet canyons. (*B*) Large drainage System 2 centered at –7.1°N, 3.2°E, characterized by minimal contributions from outlet canyons. Examples of locally sourced systems are highlighted with black squares.

Combining the global mapping of valley networks (12–15), lakes (16, 17, 22), outlet canyons (16, 17), and fluvial depositional systems (18–21) allowed us to identify regions where these systems are interconnected, forming large drainage networks (e.g., Fig. 2). In some cases, inferences of connection were made, particularly when we observe impact craters that disconnect the systems, as well as locations where there is evidence of erosion (*Materials and Methods*). We manually mapped all systems with drainage areas exceeding 10^5^ km^2^, a threshold commonly used to define large drainage systems on Earth (1). Our mapping revealed 16 large drainage systems across the surface of Mars and (Figs. 1*C* and 2 and *SI Appendix*, Figs. S1–S9 and Table S1). These systems collectively cover ~4 × 10^6^ km^2^, representing approximately 5% of Early Hesperian and older terrains (31), which span ~8 × 10^7^ km^2^ (Fig. 1*D*). However, this estimate is almost certainly conservative, as impact events alone have likely removed over 50% of valley networks (32), and additional processes such as wind erosion (33), burial, and exhumation have further obscured much of Mars’ early fluvial history (34).

**Fig. 3.**
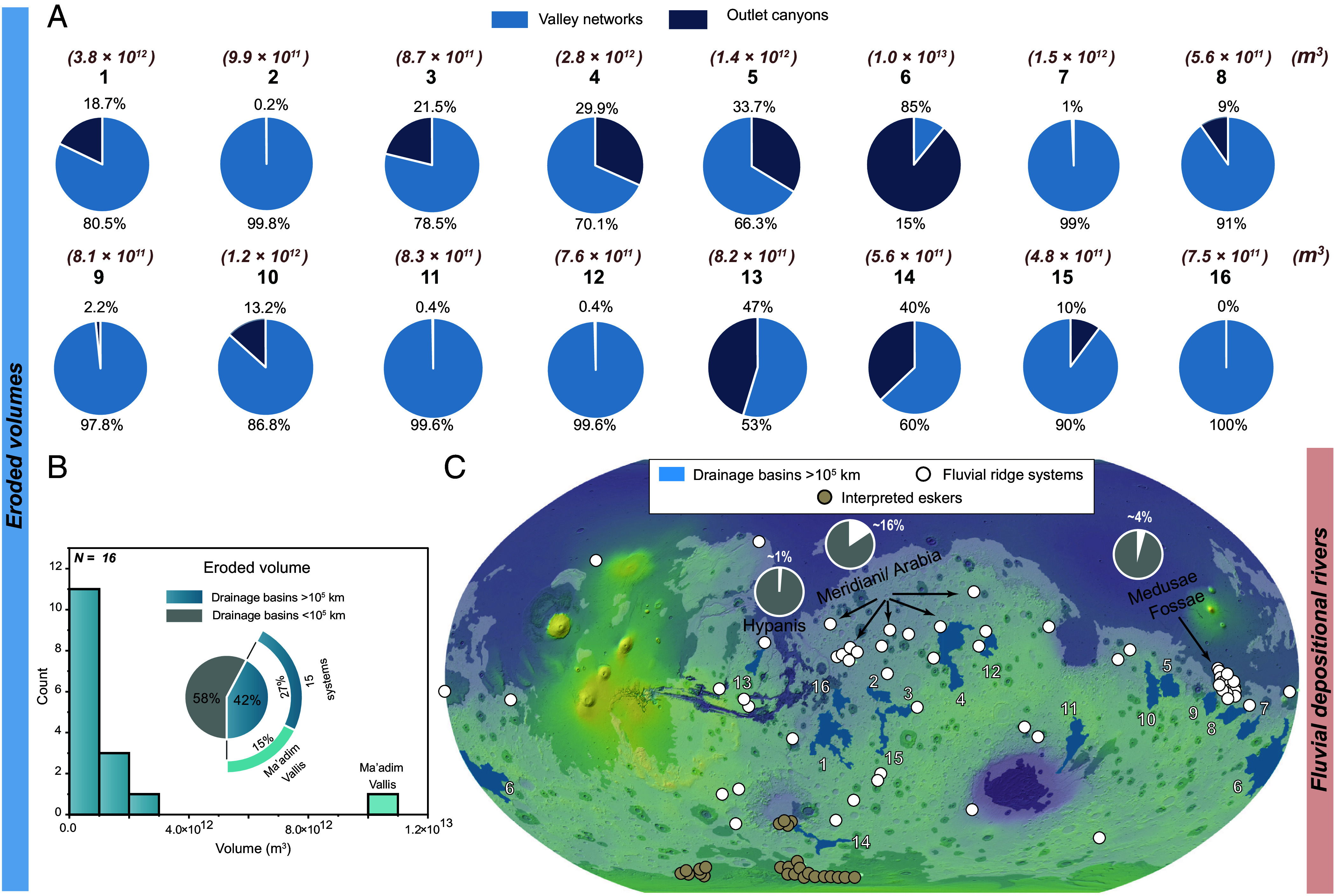
Distribution of global eroded volumes from large drainage systems and associated depositional patterns. (*A*) Relative and absolute contributions of eroded sediment from valley networks (light blue) and outlet canyons (dark blue) within each of the 16 large drainage systems. (*B*) Histogram of total eroded volumes for the 16 mapped large drainage systems. The inset pie chart illustrates their collective contribution to the total river sediment budget, with ~42% of the eroded sediment originating from these systems and ~15% from the Ma’adim Vallis system alone. (*C*) Spatial distribution of the mapped drainage systems and their connections to major depositional features, including sedimentary ridges (fluvial ridges and interpreted eskers) (17). Inset pie charts show the estimated sediment contributions to the major sedimentary basins and fan systems at Medusae Fossae (Aeolis Dorsa), Meridiani/Arabia, and Hypanis (white segments), compared to the total eroded volumes from the global valley networks (gray segments).

Another type of landform incorporated within these large drainage systems are fluvial ridge systems. These ridges are sedimentary deposits interpreted as remnants of ancient depositional river channels. Clear connections between fluvial ridges and their erosional counterparts are observed in only three of our mapped large drainage systems—two in Arabia Terra and one in Hypanis Valles (*SI Appendix*, Figs S2, S7, and S10) (17–20, 35). The remaining 13 systems do not exhibit direct links to depositional features. However, the two largest fluvial depositional complexes on Mars are situated in Aeolis Dorsa and Arabia Terra (19–21). Our mapping shows that seven large drainage systems bound the depositional region in Arabia Terra, while three bound Aeolis Dorsa (Fig. 3).

The anatomy of these large drainage systems reveals that valley networks within their boundaries have a combined length of 1.29 × 10^5^ km, representing approximately 27% of the total valley network length across Early Hesperian and older terrains (4.86 × 10^5^ km) (Fig. 1*D*). Outlet canyons, formed by lake-breach floods (16, 17), are present in all of the large drainage systems except one (System 16) (*SI Appendix*, Figs. S1–S9), with a combined length of 6.72 × 10^3^ km—accounting for approximately 49% of the global outlet canyon length (1.38 × 10^4^ km).

We further assessed why some fluvial valley segments merge to create large drainage systems while others remain isolated. To explore this, we analyzed the density of fluvial valleys and outlet canyons within both large drainage systems and regions lacking such systems. We calculated drainage density by dividing the total length of valley networks and outlet canyons by the area of both the large drainage systems and the Early Hesperian and older terrains. Our analysis reveals significant variations in drainage density. The large drainage systems are more fluvially modified, with a drainage density of 0.033 km/km^2^, whereas early Hesperian and older landscapes outside these systems exhibit a lower, globally averaged drainage density of 0.005 km/km^2^—nearly seven times lower.

### Sediment Volumes and Depositional Patterns.

As our results indicate that large drainage systems existed on early Mars, an important question arises: What was their role in the global sediment budget? Did they contribute only a small fraction of fluvial sediment, given their limited spatial extent? Or was their contribution larger in comparison to their area? To address this, we quantified the volumes of valley networks and outlet canyons within the mapped drainage systems using previously derived valley depth measurements from a progressive black top-hat (PBTH) transformation (17, 30, 36) of Mars Orbiter Laster Altimeter (MOLA) global gridded topography (8).

Our results show that the 16 mapped drainage systems collectively produced approximately 2.8 × 10^13^ m^3^ of sediment, which represents ~42% of the total estimated fluvial sediment volume on early Mars (6.7 × 10^13^ m^3^; Fig. 3) (17). The sediment volumes of individual systems range from 4.7 × 10^11^ m^3^ (0.70% of the total eroded volume of valley networks) to 1 × 10^13^ m^3^ (~15 %), with the largest contribution originating from the drainage system surrounding the Ma’adim Vallis outlet canyon (15, 17) (System 6, Fig. 3). Notably, the total sediment volume from outlet canyons within these large drainage systems is 1.16 × 10^13^ m^3^, representing nearly 41% of the total volume contributed by these large drainage systems. This suggests that outlet canyons played a major role in shaping large drainage systems, despite contributing only ~24% of the global river sediment budget on early Mars (17).

The large volume of sediment eroded from these drainage systems raises a fundamental question: Where did the sediment go? One possibility is that these systems were once connected to the largest depositional basins and fan systems on Mars, such as those located in Aeolis Dorsa and Arabia Terra (18–21, 35). Direct connections are observed in only three depositional sites—two in Arabia Terra and the Hypanis fan system (*SI Appendix*, Figs S2, S7, and S10; 18–21, 35) due to substantial influence from burial and exhumation over billions of years (32–34). We hypothesize that these systems may have fully or partially delivered sediments to these sites, a possibility that further work could confirm or refute.

To explore possible connections between large drainage systems and large sedimentary basins, we quantified the sediment volumes eroded from the seven drainage systems bounding the Meridiani/Arabia region (Systems 1, 2, 3, 4, 12, 15, and 16; Fig. 3), which together contributed ~1.05 × 10^13^ m^3^, or ~16% of the total global river sediment budget. In contrast, the three systems surrounding the Aeolis Dorsa basin (Systems 7, 8, and 9; Fig. 3) account for ~4% of the total global river sediment budget (2.9 × 10^12^ m^3^), while the Hypanis drainage system (System 13; Fig. 3) contributes ~1% (8.2 × 10^11^ m^3^). Altogether, the systems either directly connected to or bounding the largest mapped depositional basins represent ~21% of the total river sediment budget on early Mars (1.4 × 10^13^ m^3^). Although it remains unclear whether these large drainages directly correlate with the mapped depositional features (both spatially and temporally), it remains an intriguing possibility that they may have contributed to their formation.

## Discussion

**Fig. 4.**
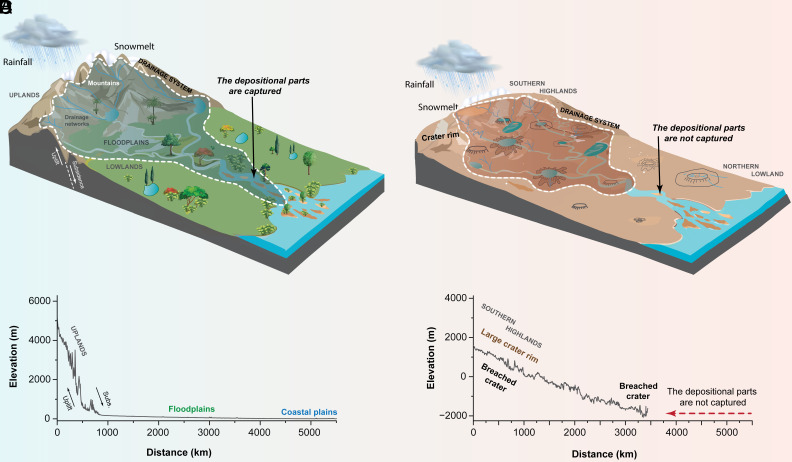
Schematic of the formation of large drainage systems on Earth (*A*) and Mars (*B*). (*C*) Longitudinal profile of the Amazon River, illustrating how the river originates from a high mountain range and transitions into vast floodplains. (*D*) Longitudinal profile of a martian drainage system, showing its origin in the southern highlands where it traversed, filled, and breached craters to form connecting outlet canyons. Both profiles are scaled with the same vertical exaggeration. The profiles were extracted from ETOPO and MOLA topographic data (*Materials and Methods*).

The existence of large drainage systems on Earth has been attributed to a combination of climate, specifically precipitation, and tectonism (9–11, 37). Tectonics elevate surface topography that serves as sediment sources, while subsidence creates vast lowlands that facilitate river development and flow toward ocean basins under climatic conditions with sufficient surface runoff (Fig. 4, 1, 11, 37). Here, we show that large drainage systems also existed on early Mars, despite the absence of plate tectonics (Figs. 1*D* and 2 and*SI Appendix*, Fig. S1–S9 and Table S1). This is perhaps unexpected given that Mars’ surface is dominated by craters, which tend to trap drainage internally and limit fluvial integration (17, 38–40). Our analysis indicates that lake-breach floods played a major role in connecting martian valley networks, ultimately leading to the development of large, integrated drainage systems (Fig. 4). Several lines of evidence support the role of lake-breach floods in the formation of large drainage systems on Mars. First, 49% of the total global length of outlet canyons—features formed by basin overflow floods (16, 17)—are located within these large drainage systems, where both initial intense overflows and/or repeated moderate discharges could have contributed to their excavation (28, 29, 41–43). Second, although large drainage systems cover only ~5% of Early Hesperian and older terrains, they are consistently associated with outlet canyons—except in a single case (System 16)—suggesting a genetic link. Third, despite their limited spatial extent, large drainage systems account for ~42% of the total eroded sediment from martian river networks (~2.8 × 10^13^ m^3^), with ~17% (1.17 × 10^13^ m^3^) out of the ~42% produced specifically by outlet canyons, comparable to the ~25% (1.66 × 10^13^ m^3^) produced by nonoutlet canyon valleys). Together, these findings indicate that large drainage systems on Mars likely formed through the integration of smaller fluvial networks via lake-breach floods. This mechanism, though not tectonically driven but rather climate-driven, was capable of producing nearly half of the total fluvial sediment on the planet from large drainage systems.

While large drainage systems existed on Mars, their limited spatial extent—covering only ~5% of Early Hesperian and older terrains—suggests that the planet’s fluvial organization was likely dominated by low-order, poorly connected networks rather than high-order, integrated systems. Our analysis supports this interpretation: The valley network density within large drainage systems is ~0.033 km/km^2^, nearly seven times higher than that of the surrounding terrain (~0.005 km/km^2^). When compared with previous work, our drainage density of ~0.033 km/km^2^ is approximately three times higher than the global estimate of 0.011 km/km^2^ reported by Hynek et al. (14) for terrains older than 3.7 Ga, and notably higher than the mean (~0.010 km/km^2^) and 90^th^ percentile (~0.025 km/km^2^) values from a global analysis of valley network geometry (40), reflecting that our measurements are confined to areas of concentrated fluvial activity. Despite covering only ~5% of ancient terrains, large drainage systems contain 27% of the global valley network length, whereas the remaining ~73% is distributed across ~95% of the Early Hesperian and older terrain, mostly in disconnected, low-density networks. This disparity in drainage densities—higher within large systems and significantly lower outside—indicates that early Mars was hydrologically dominated by small, locally sourced systems that terminated in topographic depressions such as craters (nearly ~58% of the total eroded volumes of sediments get deposited locally), rather than by large-scale integrated networks. Furthermore, while this 5% areal coverage of large drainage systems estimate is likely conservative due to resurfacing processes—including impact cratering (estimated to have removed up to 50% of valley networks) (32), wind erosion (33), burial, and exhumation (34)—even a doubling of the mapped extent would remain far below the ~44% of land area occupied by large drainage systems on Earth (Fig. 1). Taken together, our estimates of drainage density, length, and spatial extent indicate that most of Mars’ fluvial history was shaped by small, locally drained systems.

Based on our analysis, it can be extrapolated that while large drainage systems did exist on early Mars, locally sourced systems were spatially dominant. This simple understanding leads to a key question: Can the spatial distribution of sediment sinks be inferred from the drainage structure? Specifically, were eroded sediments deposited locally or transported regionally—and can these deposits be traced? Our results show that ~42% of the total eroded fluvial sediment volume on Mars originated from large drainage systems, which we hypothesize was likely routed into major depositional environments. This estimate is also consistent with the eroded volumes calculated for the 21 largest valley network systems in previous work (44). We find that large drainages bound three large sedimentary basins and fans systems—Aeolis Dorsa, Arabia Terra, and Hypanis—as key sediment sinks (18–21, 35). The large drainage systems that bound these regions together contributed ~21% of the total global river sediment budget to these basins. Although direct calculation of preserved volumes in these depositional regions is challenging due to extensive burial and exhumation (33, 34), these three sites represent likely repositories of regionally sourced sediment. Although we lack direct volume estimates for the depositional landforms themselves (e.g., fluvial ridges and fan systems), the spatial association of these landforms with major erosional sources leads us to hypothesize that a substantial portion of this eroded sediment was delivered to these sinks, particularly in regions with dense clusters of fluvial depositional landforms (Fig. 3*C*). This implies, then, that majority of the sediment produced by global valley networks (i.e., the remaining ~79% from both large and small drainages) was either removed through subsequent erosion or buried in yet-unidentified sedimentary basins, which highlights the critical role of burial and exhumation in the redistribution and long-term storage of sediments across the martian surface (17, 40, 41, 45–47). The fate of the sediment sourced from large drainage systems that do not bound large sedimentary basins thus remains uncertain. For example, the significant volume of sediment eroded from the Ma’adim Vallis system may have accumulated at its terminus, although no well-preserved depositional system is currently observed, possibly due to burial (34), subsequent erosion (33), or the northern lowlands resurfacing (48, 49). Other large drainage systems may have deposited material in large sedimentary basins such as Hellas, Argyre, or along the hemispheric dichotomy, which offer sufficient accommodation space (*SI Appendix*, Figs. S6 and S8). We suggest that some of these locations likely preserve key signals of long-distance sediment transport, potentially over hundreds to thousands of kilometers, which is also consistent with recent evidence for significant longshore transport in the northern lowland (45).

Large drainage systems on Earth, both past and present, are important landforms that transport sediments from continents to ocean basins, preserving records of tectonism, climate, and life (1–3, 50). Our identification of 16 large drainage systems on Mars poses the possibility of unexplored sedimentary basins that could serve as important repositories of ancient climates and biosignatures. This is supported by our finding that nearly half of Mars’ fluvial sediments were transported through these large drainage systems over substantial distances, suggesting that prolonged water–rock interactions across diverse geologic units may have created prime targets for biosignature exploration—an interpretation consistent with other lines of geologic evidence for prolonged water–rock interaction (51–54). These basins may have been eroded or buried over long periods due to alternating geological processes such as sediment deposition, burial, and wind-driven exhumation (32–34). Our results further suggest that the formation of large drainage systems on other planetary bodies does not require a specific tectonic setting, only some collection of processes capable of generating topography and a hydroclimate sufficient to first develop and then integrate channels into dense river networks.

## Materials and Methods

### Mapping Large Drainage Systems on Mars.

For our mapping, we used previously published global datasets of valley networks (14, 17), lakes (17, 22), and fluvial ridge systems (19). We combined these datasets and conservatively drew polygons that outline the extent of individual drainage systems. Unlike on Earth—where fluvial systems typically consist of a catchment area dissected by drainage networks, followed by transport zones and depositional sinks such as floodplains and coastal plains (50)—limited preservation of ancient martian fluvial systems means downstream depositional sinks are rarely connected to upstream sources. This difference, expected due to variations in topography and limited preservation over >3.5 Gyr, is characterized by valley networks that drain into craters (some interpreted as ancient lakes), outlet canyons formed by lake-breach floods, and, in a few cases, downstream depositional rivers expressed as sedimentary ridges (14–21). All drainage system boundaries were manually delineated in ArcGIS Pro (55), guided by topographic contrasts between surrounding terrain and the water-formed landforms included in our mapping using Mars Orbiter Laser Altimeter (MOLA) global gridded data (8).

Before mapping large drainage systems, we established a set of criteria to guide the delineation of the bounding polygons. First, we reviewed previous studies to identify regions with high densities of mapped valley networks, which helped systematically locate fluvially dissected “hotspots.” Second, we examined topographic and image data [Mars Reconnaissance Orbiter and its Context Camera (CTX)] (55, 56) to assess the connectivity between valley networks and mapped lakes. Third, when lakes exhibited clear breach points leading to outlet canyons, we included those features as part of the drainage system extent. In three systems (two in Arabia Terra and Hypanis Valles), we also observed direct connections between erosional components (valley networks and outlet canyons) and downstream depositional features (fluvial ridge systems and fans), which were incorporated into the mapped boundaries.

Using this approach, we initially mapped 19 large drainage systems. However, upon measuring their areas, we found that three of the systems were smaller than 10^5^ km^2^—the threshold commonly used on Earth to define large drainage systems (1). As a result, we excluded these three systems from further analysis (*SI Appendix*, Table S2).

Unlike previous studies (57), we did not apply automated watershed delineation, as such methods tend to overestimate drainage areas and mismatch the observed landforms and the topographic structures. Mars’ topography has undergone substantial changes over billions of years due to the growth of the Tharsis rise (58), widespread wind erosion and exhumation following deep burial (33, 34), and impact cratering (32). These processes have likely altered or obscured the original surface topography, making manual delineation based on geomorphic and topographic context a more reliable approach.

### Quantifying Eroded Volumes.

After mapping the large drainage systems, we aimed to assess the volume of eroded sediment from these systems relative to total eroded volumes from valley networks and outlet canyons. To achieve this, we used global datasets of valley network and outlet canyon depths, previously extracted and calculated from the MOLA Mission Experiment Gridded Data Record (MEGDR) digital elevation model using a Progressive Black Top-Hat (PBTH) transformation (17, 35, 36). We then calculated the geodesic area for each pixel in the resulting valley depth raster and computed the eroded sediment volumes for each of the 16 mapped drainage systems using zonal statistics analysis in ArcGIS Pro (52). We performed these calculations separately for valley networks and outlet canyons to quantify the relative contribution of each landform, which helped provide insights for evaluating hypotheses on the mechanisms responsible for forming large drainage systems on Mars.

### Additional Data Used for Analysis.

To provide terrestrial context, we first compiled the global distribution of large drainage systems on Earth (Fig. 1 *A* and *C*). Using a global dataset of rivers draining into the oceans from Milliman and Farnsworth (5), we filtered rivers with drainage areas exceeding 10^5^ km^2^—the commonly accepted threshold for defining large rivers (1). This yielded 91 rivers, for which we extracted data on drainage area, sediment fluxes, and main channel length to illustrate the significance of large rivers on Earth. One limitation to our approach is that sediment fluxes may be underestimated due to anthropogenic influences, including damming. However, this uncertainty is not critical to our study’s primary conclusions.

In Fig. 4 *C* and *D*, we generated longitudinal profiles for representative systems on both Earth and Mars to illustrate the drivers of importance for the formation of large drainage systems. The terrestrial example is the Amazon River, derived from ETOPO global topographic data. The martian example corresponds to System 1 (–22.4°N, –17.7°E), shown in Fig. 2*A*, with the profile generated from MOLA topography. The elevation and distance data used to construct these profiles are provided in Datasets S1 and S2.

### Potential Limitations and Uncertainties.

While our findings offer insights into the development of fluvial landscapes on Mars, the extent of past habitable environments, and sediment transport dynamics, it is important to acknowledge key limitations and sources of uncertainty. First, the drainage systems were mapped conservatively to minimize potential overestimation of drainage area and, consequently, eroded sediment volumes. Second, preservation biases likely obscure a significant portion of the original landscape signal, particularly the valley networks. Several geological processes may have contributed to this loss, including impact cratering (32), kilometer-scale wind erosion and exhumation (33), burial (34), and surface deformation associated with construction of Tharsis rise (49, 58). Although the individual contribution of each process remains uncertain, recent work has estimated that impact cratering alone may have erased up to ~50% of valley networks (8). If the other major processes exerted comparable influence, the total uncertainty in mapped drainage area—and possibly associated volumes—could be as high as a factor of two. Future work to isolate and quantify the effects of these resurfacing processes will be critical for improving our understanding of the origin, evolution, and preservation of ancient martian fluvial systems.

An additional uncertainty arises from the difficulty of directly linking erosional and depositional rivers, as the flow directions of the depositional systems remain unconstrained (59), as do precise system ages. Further work reconstructing the source-to-sink pathways of fluvial depositional systems is essential to determine whether they represent the downstream continuation of the mapped erosional drainage systems.

One last limitation is that, although valley networks provide important insights into runoff-driven erosion, they likely represent only a modest fraction of total erosion and sediment redistribution in the martian highlands. Older intracrater erosion and sedimentation, along with the infilling of internally drained basins, probably contributed far greater volumes of material (60, 61). However, our results provide direct insights into the era of peak valley network formation and the associated valley incision.

## Supplementary Material

Appendix 01 (PDF)

Dataset S01 (XLSX)

Dataset S02 (XLSX)

## Data Availability

All study data are included in the article and/or supporting information. The mapped large drainage systems and the Fig. 4 profiles are available in the Texas Data Repository (62), and the dataset of eroded volumes for valley networks and outlet canyons is available at ref. 63.
