# Supplementary material for: Large drainage systems produced half of Mars’ ancient river sediment
Source: Proc Natl Acad Sci U S A. 2025 Nov 24;122(48):e2514527122. doi: 10.1073/pnas.2514527122 (PMC12685115; doi:10.1073/pnas.2514527122)
Supplement: Supplementary file 1 — Appendix 01 (PDF) [file pnas.2514527122.sapp.pdf]

Supporting Information for

Large drainage systems produced half of Mars' ancient river sediment

Abdallah S. Zaki[1,2]*, Timothy A. Goudge[1,2], David Mohrig[1]

[1]Department of Earth and Planetary Sciences, Jackson School of Geosciences, The University of Texas at Austin, Austin, TX, USA
[2]Center for Planetary Systems Habitability, The University of Texas at Austin, Austin, TX, USA
*Corresponding author: Abdallah S. Zaki

This PDF file includes:

Figures S1 to S10
Tables S1 to S2

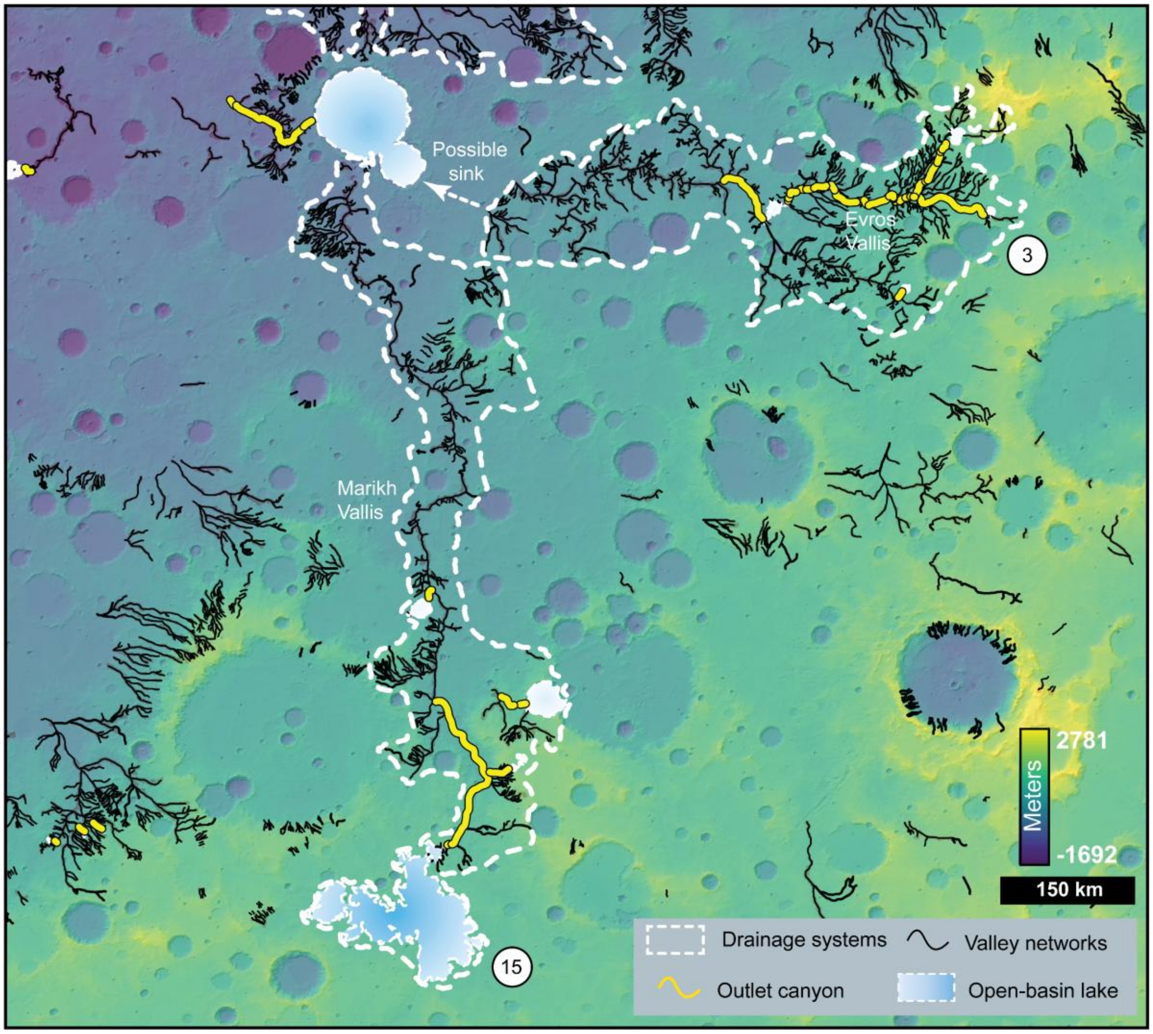

Fig. S1. Mapping of Systems 3 and 15 at (11.69°E, 12.81°S) and (4.35°E, 20.88°S), respectively. Dashed white outlines represent drainage boundaries encompassing valley networks (black), lakes (shaded blue), and outlet canyons (yellow) formed by lake breaches. The background map is derived from Mars Orbiter Laser Altimeter (MOLA) topography.

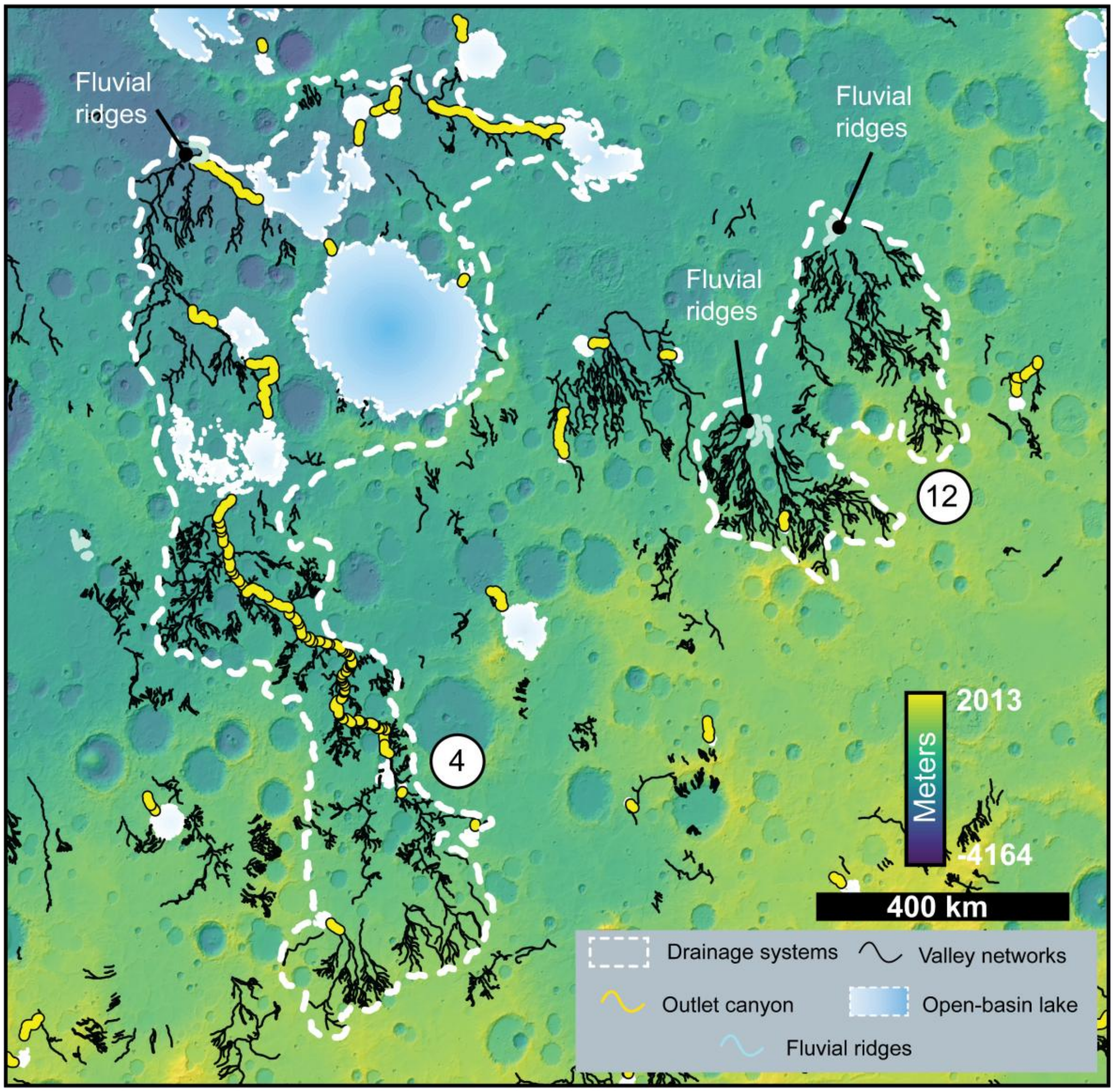

Fig. S2. Mapping of Systems 4 and 12 at (33.77°E, 9.7°N) and (49.45°E, 11.48°N), respectively. Dashed white outlines represent drainage boundaries encompassing valley networks (black), lakes (shaded blue), outlet canyons (yellow) formed by lake breaches, and fluvial ridge systems (light blue polylines). The background map is derived from Mars Orbiter Laser Altimeter (MOLA) topography.

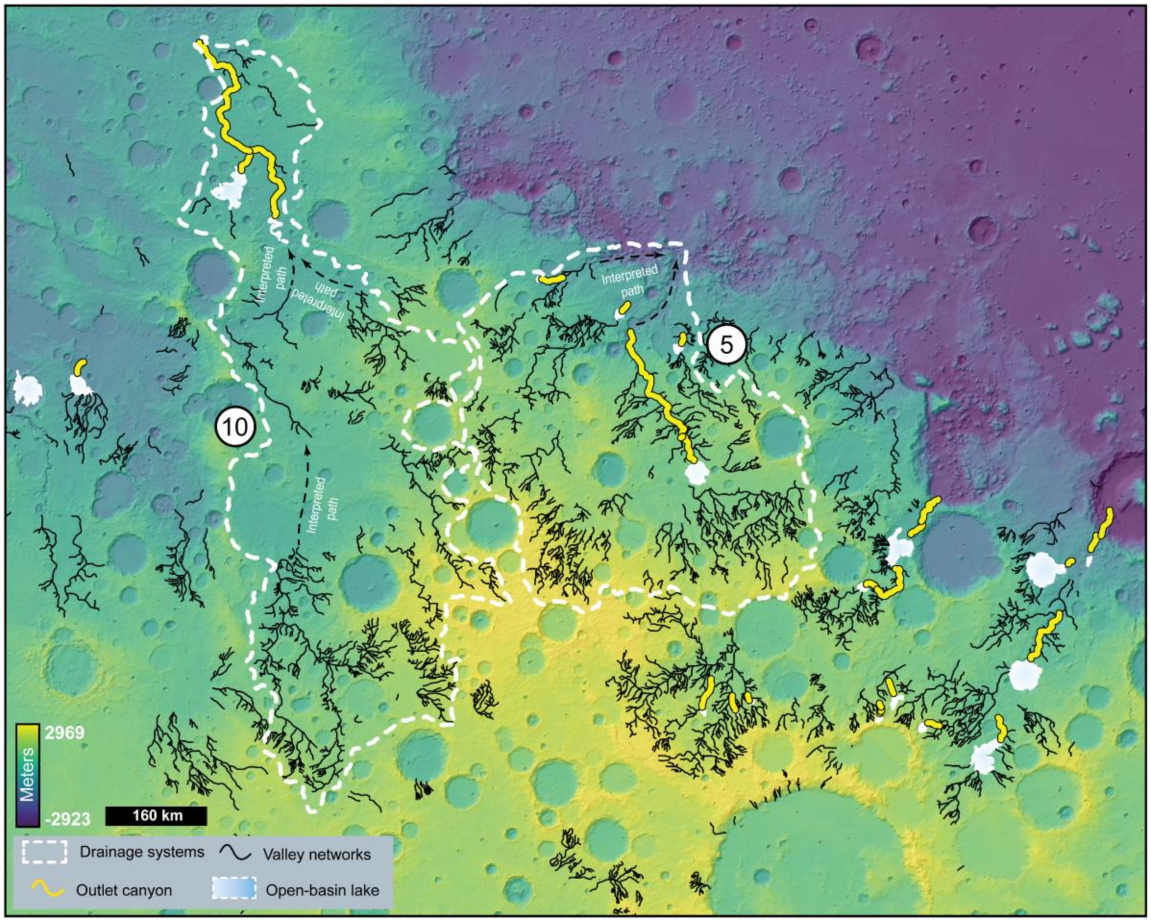

Fig. S3. Mapping of Systems 5 and 10 at (125.49°E, 3.81°S) and (118.20°E, 4.13°S), respectively. Doshed white outlines represent drainage boundaries encompassing valley networks (black), lakes (shaded blue), and outlet canyons (yellow) formed by lake breaches. The background map is derived from Mars Orbiter Laser Altimeter (MOLA) topography.

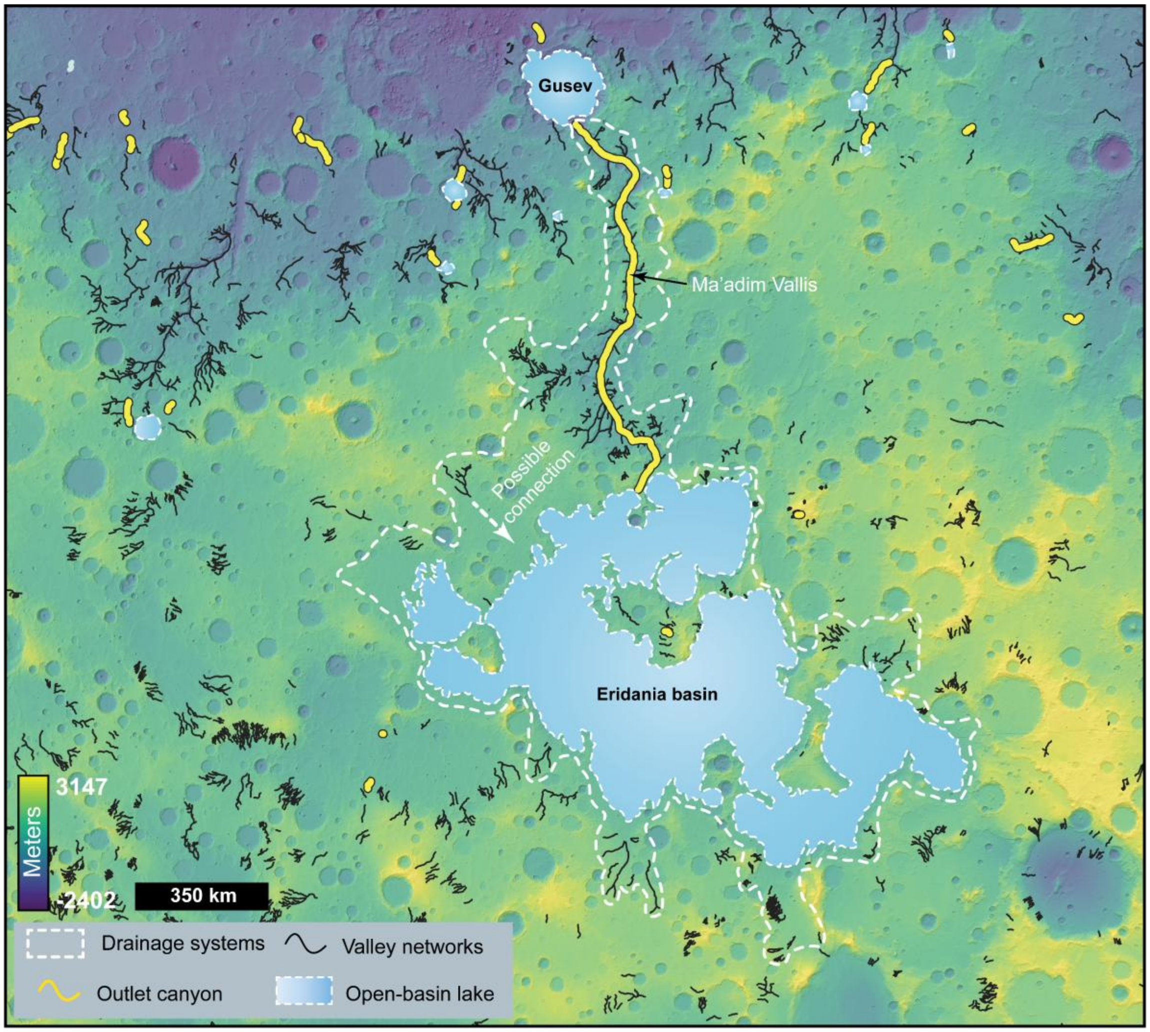

Fig. S4. Mapping of System 6 (Ma'adim Vallis) at (29.9°E, 34.23°S). Dashed white outline represents drainage boundaries encompassing valley networks (black), lakes (shaded blue), and outlet canyons (yellow) formed by lake breaches. The background map is derived from Mars Orbiter Laser Altimeter (MOLA) topography.

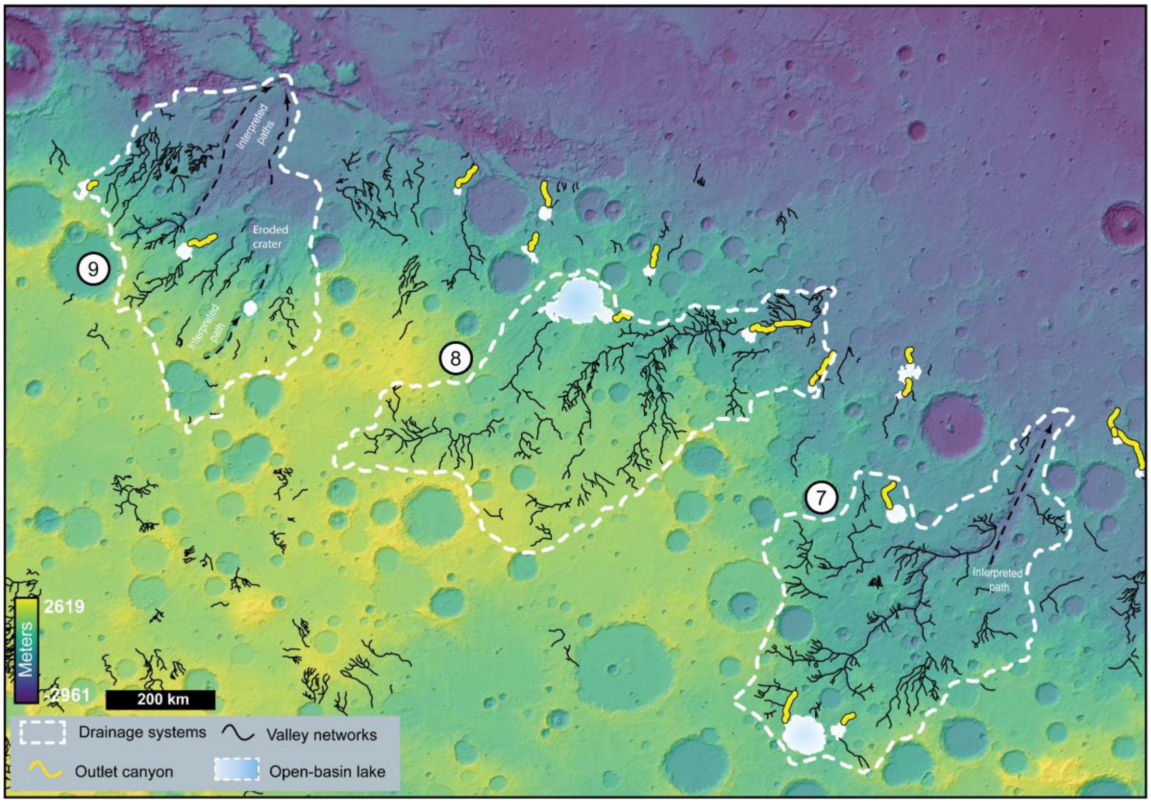

Fig. S5. Mapping of Systems 7,8, and 9 at (161.05°E, 19.53°S), (152.95°E, 14.20°S), and (143.15°E, 9.96°S), respectively. Dashed white outlines represent drainage boundaries encompassing valley networks (black), lakes (shaded blue), and outlet canyons (yellow) formed by lake breaches. The background map is derived from Mars Orbiter Laser Altimeter (MOLA) topography.

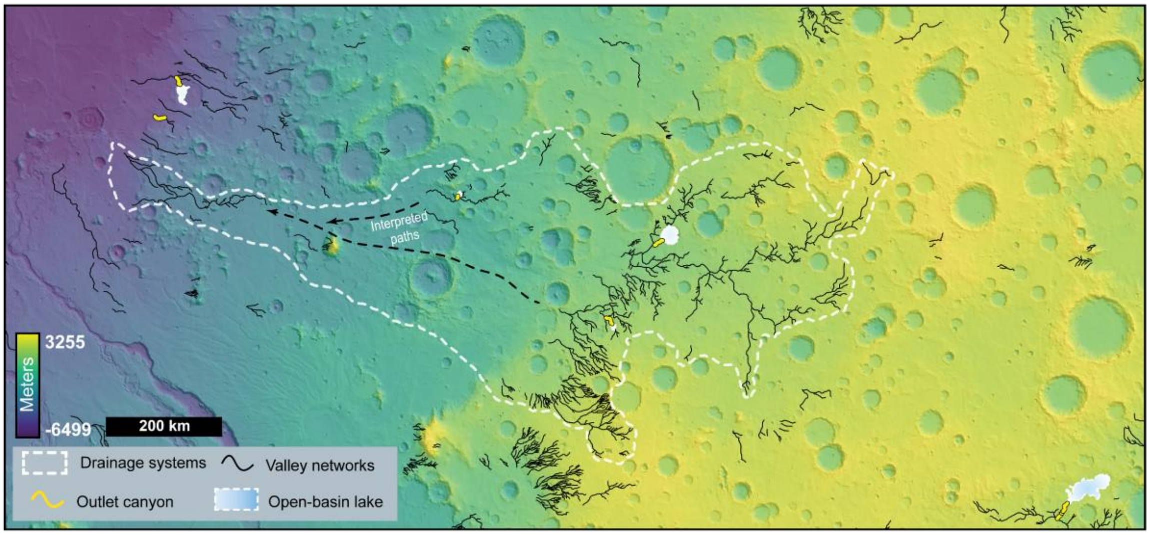

Fig. S6. Mapping of System 11 at (87.52°E, 24.19°S). Dashed white outlines represent drainage boundaries encompassing valley networks (black), lakes (shaded blue), and outlet canyons (yellow) formed by lake breaches. The background map is derived from Mars Orbiter Laser Altimeter (MOLA) topography.

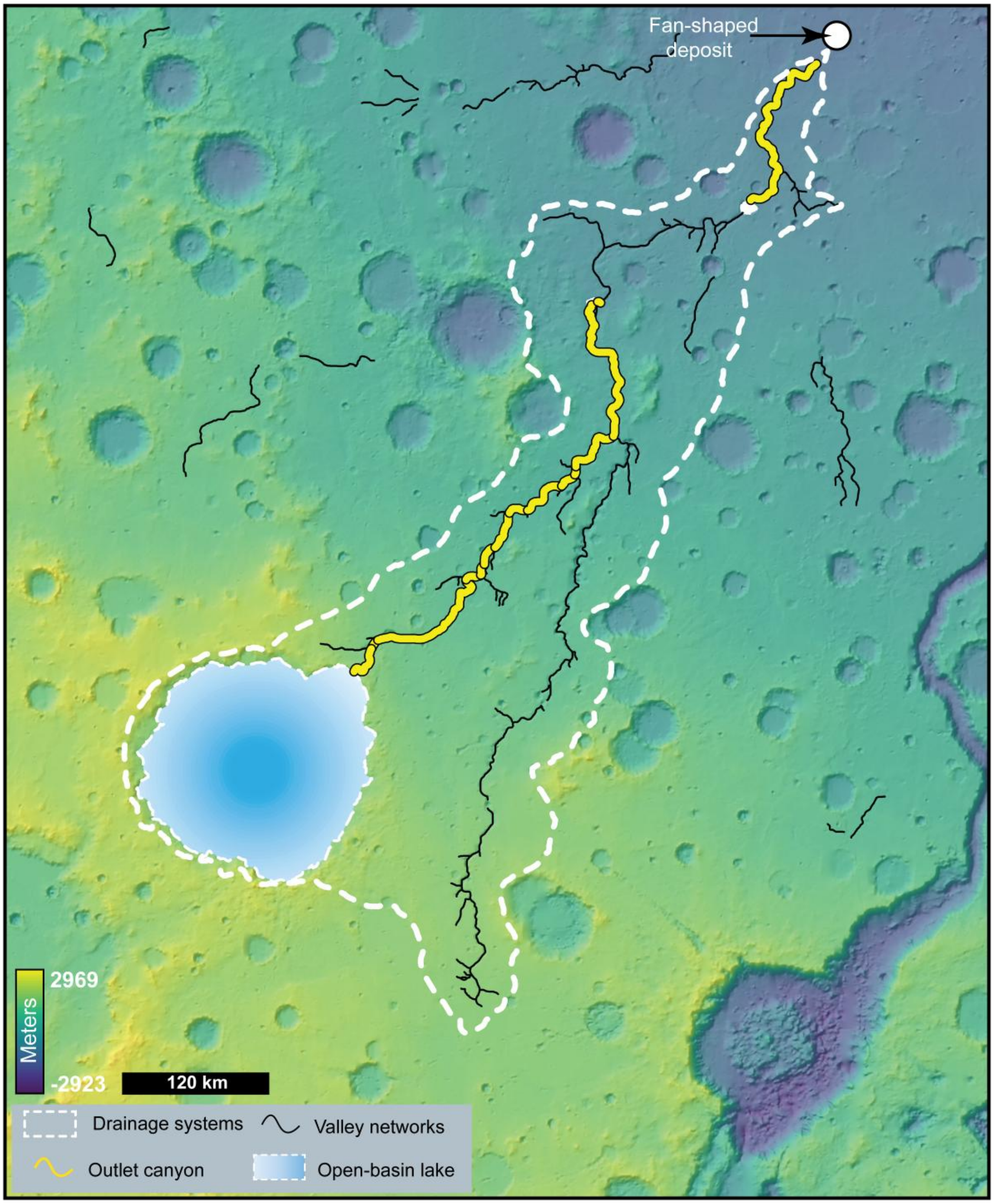

Fig. S7. Mapping of System 13 (Hypanis) at (49.25°W, 5.10°N). Dashed white outlines represent drainage boundaries encompassing valley networks (black), lakes (shaded blue), and outlet canyons (yellow) formed by lake breaches. At the system terminus, an inverted fan-shaped deposit is present (open white circle). The background map is derived from Mars Orbiter Laser Altimeter (MOLA) topography.

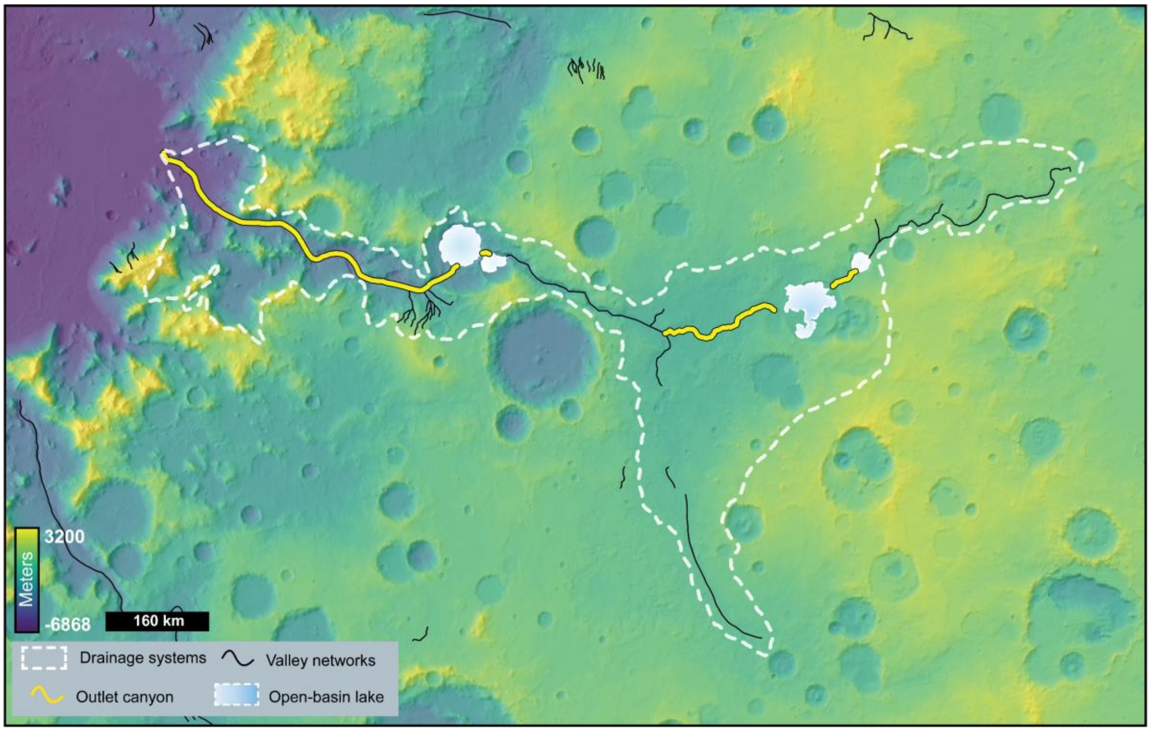

Fig. S8. Mapping of System 14 at (27.66°W, 62.19°S). Dashed white outlines represent drainage boundaries encompassing valley networks (black), lakes (shaded blue), and outlet canyons (yellow) formed by lake breaches. The background map is derived from Mars Orbiter Laser Altimeter (MOLA) topography.

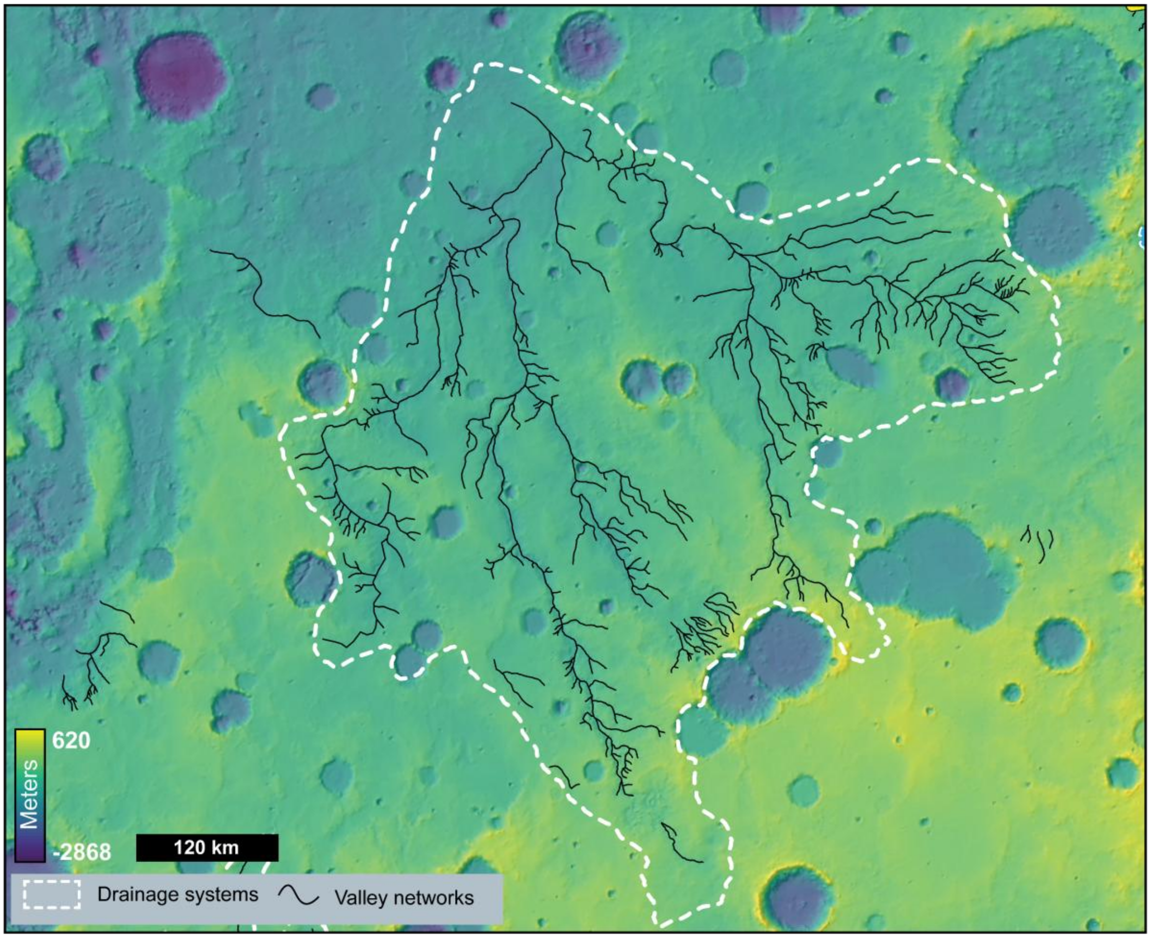

Fig. S9. Mapping of System 16 at (13.31°W, 9.85°S). Dashed white outlines represent drainage boundaries encompassing valley networks (black). The background map is derived from Mars Orbiter Laser Altimeter (MOLA) topography.

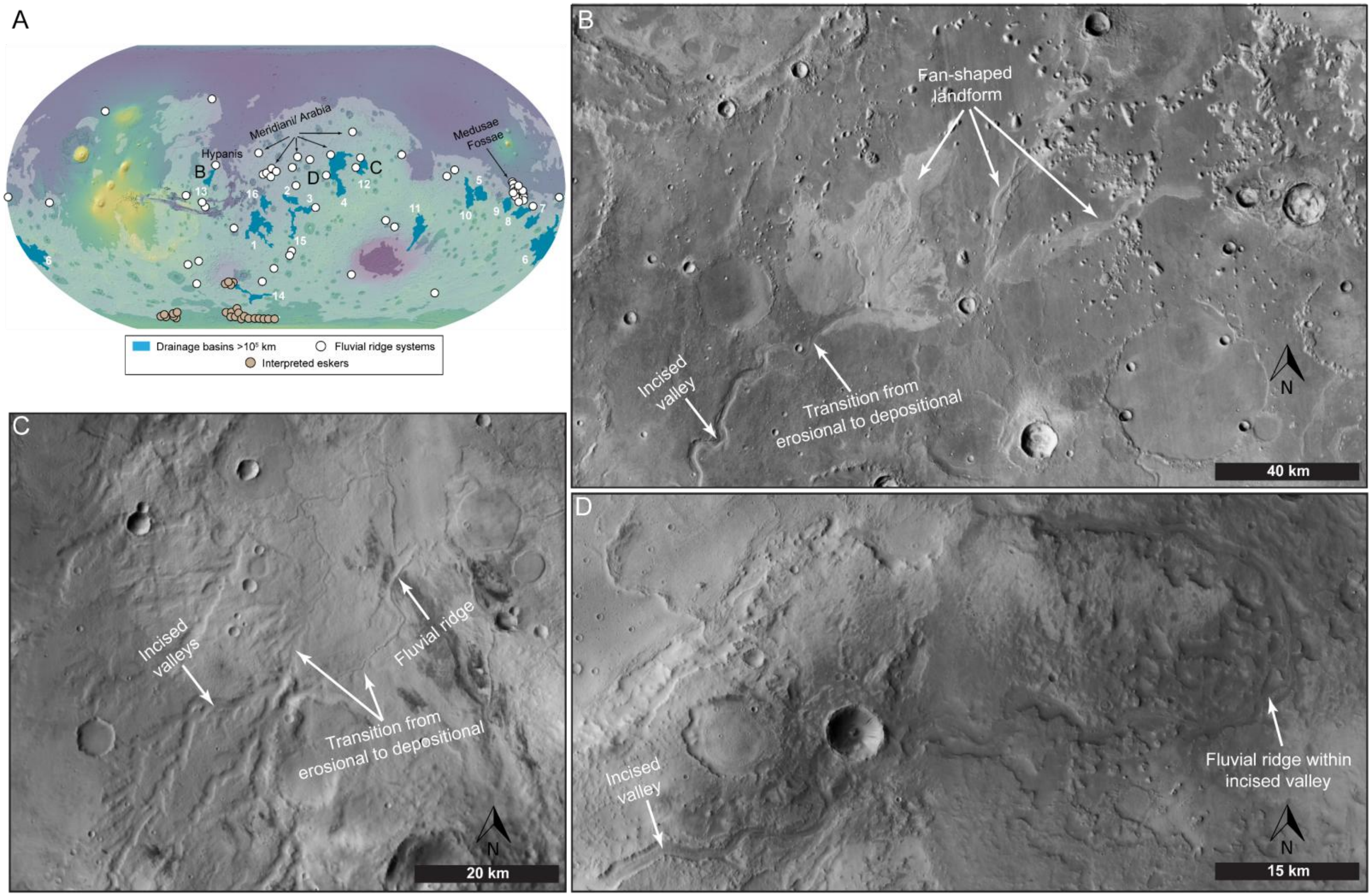

Fig. S10. (A) Global map of large drainage systems on Mars and their spatial relationship to depositional features. (B–D) CTX image mosaics showing three examples of transitions from erosional systems—marked by incised channels and valleys—to depositional features, including fluvial ridges and fan-shaped landforms, in Hypanis and Arabia Terra (*1*).

Table S1. Characteristics of drainage systems, including coordinates, area, volumes of valley networks and outlet canyons, and lake areas. The grey row highlights System 6 (Ma'adim Vallis).

System ID	Longitude	Latitude	Area (km^2)	Outlet canyon volumes (m^3)	Valley network volumes (m^3)	Total drainage system volume (m^3)	Lake areas (km^2)
1	-17.715	-22.4133	483332	7.1606E+11	3.11139E+12	3.82745E+12	18898.11574
2	3.203322	-7.11739	121586.1	1596048204	9.884E+11	9.89996E+11	722.1512706
3	11.69669	-12.8133	111895.6	1.87405E+11	6.83639E+11	8.71045E+11	818.1423754
4	33.77179	9.703046	571091.4	8.34079E+11	1.95886E+12	2.79294E+12	100313.3561
5	125.4917	-3.8115	188671.2	4.83925E+11	9.53038E+11	1.43696E+12	983.5053842
6	29.90007	-34.2358	995088	8.55795E+12	1.50478E+12	1.00627E+13	628297.9411
7	161.0571	-19.5358	145173.3	14341021557	1.48648E+12	1.50082E+12	3871.47334
8	152.9534	-14.2055	161490.8	51102243842	5.13604E+11	5.64706E+11	5204.139543
9	143.1574	-9.96374	127318	17619521591	7.87599E+11	8.05219E+11	875.5588217
10	118.2053	-4.13882	235210	1.59849E+11	1.05567E+12	1.21552E+12	1390.714895
11	87.52087	-24.1972	286257.4	2897339859	8.22302E+11	8.252E+11	739.2755925
12	49.45554	11.48054	155842.6	3280799894	7.56297E+11	7.59578E+11	48.58221567
13	-49.2563	5.106933	104020.3	3.86864E+11	4.36516E+11	8.2338E+11	18912.08349
14	-27.6654	-62.1906	113382.6	2.22653E+11	3.35952E+11	5.58604E+11	6935.266273
15	4.355009	-20.8873	120801.3	49902718101	4.27339E+11	4.77242E+11	15933.60536
16	-13.3178	-9.85114	102584.2	0	7.4632E+11	7.4632E+11	0

Table S2. Locations and areas of the systems that were not included in our analysis because they did not exceed the area threshold (10^5 km²).

System ID	Longitude	Latitude	Area (Km^2)
19	134.468989°	-9.307930°	67,316
20	77.691130°	-22.762805°	37,022
21	14.462490°	36.222860°	65,772

References

1. J. L. Dickson, B. L. Ehlmann, L. Kerber, C. I. Fassett, The global context camera (CTX) mosaic of Mars: a product of information-preserving image data processing. *Earth Space Sci.* **11**, (2024).
